# Association between dietary zinc intake and *Helicobacter pylori* seropositivity in US adults: National Health and Nutrition Examination Survey

**DOI:** 10.3389/fnut.2023.1243908

**Published:** 2023-09-21

**Authors:** Kai Zhang, Yu Han, Fangming Gu, Zhaoxuan Gu, JiaYu Zhao, Jianguo Chen, Bowen Chen, Min Gao, Zhengyan Hou, Xiaoqi Yu, Tianyi Cai, Yafang Gao, Rui Hu, Jinyu Xie, Tianzhou Liu

**Affiliations:** ^1^Department of Cardiovascular Surgery, Second Hospital of Jilin University, Changchun, China; ^2^Department of Ophthalmology, First Hospital of Jilin University, Changchun, China; ^3^Bethune First College of Clinical Medicine, Jilin University, Changchun, China; ^4^Department of Cancer Center, The First Hospital of Jilin University, Changchun, China; ^5^Bethune Second School of Clinical Medicine, Jilin University, Changchun, China; ^6^Bethune Third College of Clinical Medicine, Jilin University, Changchun, China; ^7^Department of Gastrointestinal Surgery, The Second Hospital of Jilin University, Changchun, China

**Keywords:** zinc intake, *Helicobacter pylori*, association, generalized additive model, subgroup analysis

## Abstract

**Purpose:**

*Helicobacter pylori* infection is a well-established etiological factor for gastric inflammation and a significant risk factor for the development of gastric cancer. However, the precise relationship between dietary zinc intake and seropositivity for *Helicobacter pylori* remains uncertain.

**Methods:**

This cross-sectional observational study utilized data from the United States National Health and Nutrition Examination Survey conducted between 1999 and 2000. The study cohort comprised 2,884 adults aged 20 years or older who provided comprehensive 24-h dietary recall data. The presence of *Helicobacter pylori* infection was confirmed using serum analysis and lgG protein enzyme-linked immunosorbent assay (ELISA). Multivariable logistic regression models and generalized additive model (GAM) were employed to explore the potential association between dietary zinc intake and *Helicobacter pylori* seropositivity.

**Results:**

Additionally, subgroup analysis was performed to evaluate the robustness of the primary findings. Of the 1,281 participants, 47.8% were male and the average age was 49.5 years. In the fully adjusted model, a statistically significant inverse association between dietary zinc intake and *Helicobacter pylori* seropositivity was observed [quartile variable, Q4 vs. Q1, odds ratio (OR): 0.72, 95% confidence interval (CI): 0.57–0.91, *p* = 0.007]. Furthermore, the relationship between dietary zinc intake and *Helicobacter pylori* seropositivity exhibited an L-shaped pattern, indicating a saturation effect. The results of sensitivity analysis remained consistent and reliable.

**Conclusion:**

Therefore, this study suggests that higher dietary zinc intake may be associated with a lower prevalence of *Helicobacter pylori* seropositivity. Notably, this association follows an L-shaped pattern, with a threshold point estimated at 24.925 mg/day.

## Introduction

*Helicobacter pylori* (*H. pylori*) is a bacterial pathogen that establishes colonization within the gastric mucosa, thereby inducing persistent inflammation and gastropathy (1). *H. pylori* infection has been implicated in the etiology of diverse gastrointestinal disorders, including chronic gastritis, gastric ulcer, gastric cancer, and mucosa-associated lymphoid tissue lymphoma (2–4). The standard therapeutic regimen for *H. pylori* eradication, known as triple therapy, involves administration of a proton pump inhibitor (PPI), amoxicillin, and clarithromycin (5). However, the emergence of drug-resistant strains of *H. pylori* has resulted in diminished efficacy of conventional triple therapy, as evidenced by eradication rates as low as 50% reported in certain investigations (6). In recent times, growing attention has been directed towards exploring the correlation between *Helicobacter pylori* seropositivity and micronutrient intake, with specific emphasis on trace elements such as zinc, iron, calcium, and copper present in the diet. These trace elements hold vital significance for human health, and their insufficiency may engender detrimental consequences on overall well-being (7).

Zinc, an essential micronutrient, plays a pivotal role in various biological processes encompassing DNA replication, recombination, repair, protein synthesis, gene regulation, and cell division. Inadequate zinc levels can impede the growth of both humans and animals, underscoring the importance of comprehending its impact on cellular mechanisms (8–10). Furthermore, zinc contributes to wound healing, DNA repair, and protection against oxidative damage (11, 12). Previous studies indicate that deficiencies in zinc, iron, and vitamin C heighten vulnerability to oxidative damage and *H. pylori* infection (13). *In vitro* and *in vivo* research has demonstrated the favorable effects of zinc linolenate, a novel selective antibiotic against *H. pylori* (14). Additionally, pH-sensitive organometallic structures in hydrogen-producing nanoparticles have shown potential in improving the gastric mucosal environment and inhibiting *Helicobacter pylori* through zinc and hydrogen degradation (15). Although previous studies have explored the association between *Helicobacter pylori* seropositivity and zinc intake at a cellular and animal level, epidemiological data on this relationship among adults are limited and inconsistent (16–18). To delve deeper into this matter, we conducted an analysis utilizing data from the 1999–2000 National Health and Nutrition Examination Survey (NHANES), which included 2,884 participants aged 20 years or older (19). Our objective was to investigate the association between dietary zinc intake and *Helicobacter pylori* seropositivity.

## Participants and methods

### Data sources and study population

The NHANES, a nationally representative cross-sectional study, employs a stratified multistage probability and oversampling design to ensure accurate representation of the noninstitutionalized civilian population of the United States (20). Biennial data releases from the study consist of participants who collectively represent approximately 50,000 US citizens. The administration of standardized interviews and examinations is carried out by trained staff, including physicians, dentists, health technologists, interviewers, and laboratory technicians (21). Comprehensive information regarding the NHANES study design, recruitment, procedures, and demographic characteristics can be found on the CDC website^1^ (22). In summary, the NHANES study employed a four-stage design with oversampling of specific subgroups to enhance precision, and an advanced computer system was used for data collection and processing. The survey findings play a crucial role in determining disease prevalence and associated risk factors.

**Figure 1:**
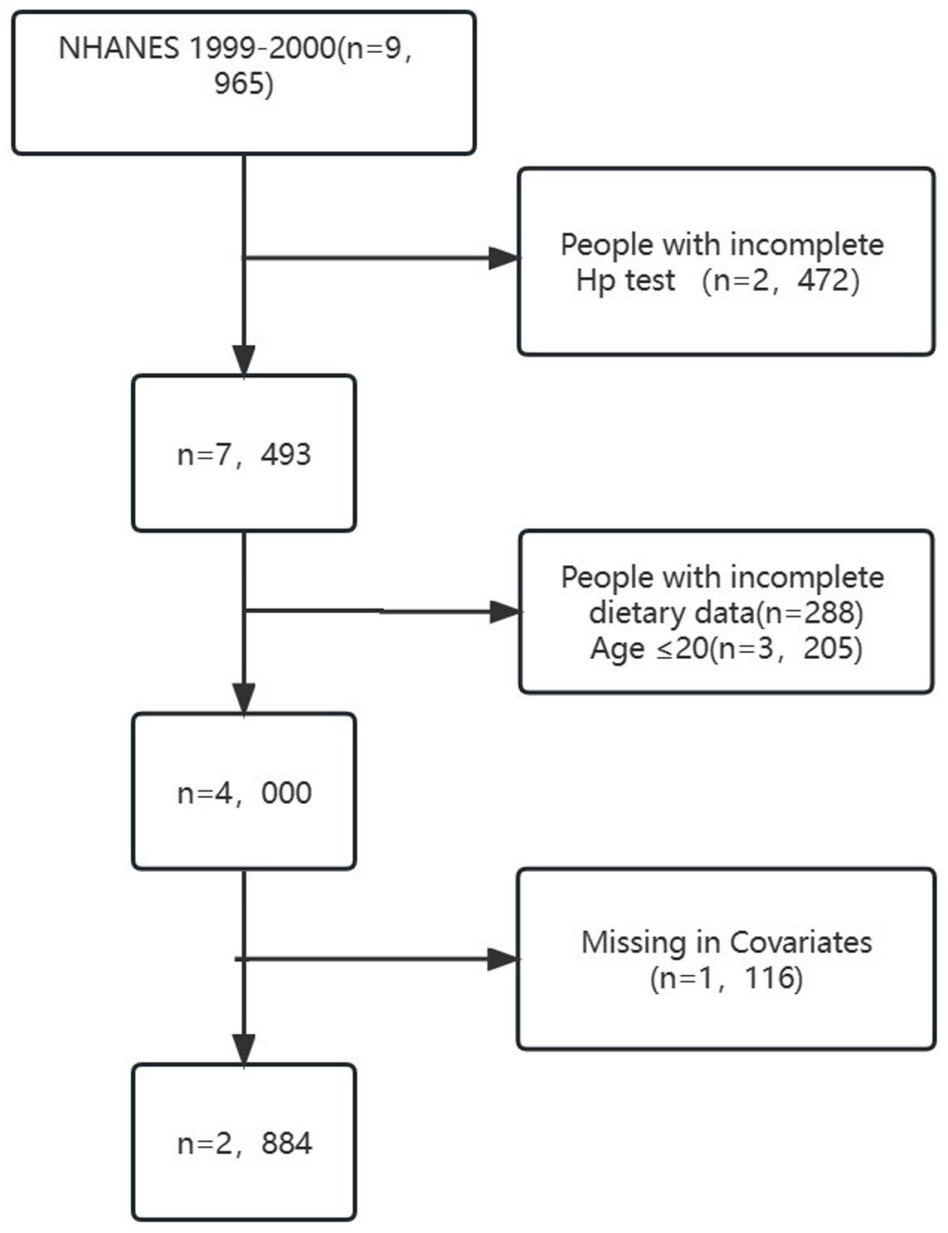
Flowchart detailing the selection process for patients included in this analysis.

The study sample consisted of 2,884 individuals aged 20 years or older, as depicted in Figure 1. Exclusion criteria led to a reduction in the sample size by 2,472 due to incomplete LBXHP1 data. Additionally, participants under the age of 20 years (*N* = 3,205) and those with incomplete dietary data (288) were excluded. Furthermore, individuals with missing information on covariates were also excluded, resulting in an additional reduction in sample size by 1,116. Specifically, there are 1,116 patients of missing covariates in our dataset, encompassing various variables: 10 patients pertain to missing education levels, 448 to missing marital status, 478 to missing Poverty inclusion ratio, 24 to missing Body mass index, 4 to missing smoke status, 114 to missing alpha status, 1 to missing diabetes status, 20 to missing white protein levels, 7 to missing Heart failure, 9 to missing Coronary disease, 9 to missing Angina, and 3 to missing Heart attack. The process of identification, screening, and inclusion for the study is presented in Figure 1. NHANES received approval from the NCHS Research Ethics Review Board, and written informed consent was provided by all participants (23). All procedures adhered to the guidelines of the Declaration of Helsinki and Strengthening the Reporting of Observational Studies in Epidemiology (STROBE).

### Dietary zinc intake

Dietary intake interviews were conducted at the NHANES mobile examination clinics through in-person sessions. To gather dietary intake data, participants of NHANES underwent two 24-h dietary recalls (24, 25). The initial recall interview occurred face-to-face at the Mobile Examination Center (MEC), administered by trained interviewers. The second interview took place via telephone or mail within a period of 3 to 10 days. The dietary assessments were based on the average of these two recalls (26). As per NHANES analytic guidelines, the combined intake of dietary zinc and zinc supplementation was considered as the total zinc intake. The analysis of zinc intake involved examining it as both continuous and categorical variables (Q1–Q4). Zinc intake quartiles (Q1–Q4) were determined by dividing the zinc intake distribution into four parts, reflecting low to high intake levels. The range of quartiles (Q1–Q4) are as follows: Q1 (<5.97) Q2 (5.97–8.92) Q3 (8. 92–13.16) Q4 (>13.16).

### *Helicobacter pylori* antibody measurement

Wampole Laboratories, located in Cranbury, NJ, employed a *Helicobacter pylori* IgG enzyme-linked immunosorbent assay (ELISA) (27) for the assessment of the immune status ratio (ISR). The ISR was calculated as the quotient of the mean optical density (OD) obtained from the test samples and the mean cutoff value (28). The *H. pylori* IgG antibodies present in human serum were identified and assessed in a qualitative manner using the *H. pylori* IgG enzyme-linked immunosorbent assay (ELISA) developed by Ratio Diagnostics, Frankfurt, Germany. The determination of *H. pylori* status relied on specified *H. pylori* antibody titers (29). Within the context of this study, the *H. pylori* infection status was determined based on the serum anti-*H. pylori* IgG level, whereby a value exceeding 0.9 for *Helicobacter pylori* lgG was regarded as indicative of a positive infection.

### Covariates

The study considered several potential confounders based on existing literature and clinical judgment. These included age, gender, marital status, poverty-income ratio (PIR), education, body mass index (BMI), cardiovascular disease (heart failure, coronary heart disease, angina, and heart attack), smoking, alcohol consumption, diabetes, and serum indicators (CRP, albumin, total cholesterol, and creatinine). Age was analyzed as a continuous variable, and gender was analyzed as a categorical variable with male and female being the two categories considered. Marital status was divided into living alone and married or living with a partner (30). Socioeconomic status was measured using the PIR, which was divided into low (PIR < 1.3) and middle-high (PIR ≥ 1.3) groups based on the relationship between PIR and 1.3. Education level was divided into below high school and above high school. BMI was categorized into normal weight group (BMI 18.5–24.9 kg/m^2^), overweight group (BMI 25–29.9 kg/m^2^), and obesity group (BMI ≥ 30 kg/m^2^) (31). Smoking status was categorized into non-smoking and smoking, and alcohol use was categorized into non-alcohol use and alcohol use. To assess diabetes, participants were asked, “Have you ever been diagnosed with diabetes or high blood sugar?” (32), and those who answered “yes” were considered diabetic. The presence of cardiovascular disease was determined by patient-reported history of heart failure, coronary heart disease, angina, or heart attack (33). Laboratory measures included CRP, albumin, total cholesterol, and creatinine.

### Statistical analysis

The data in this study were classified into two categories: continuous and categorical variables. Continuous variables were further categorized based on their distribution normality. Normally distributed continuous variables were presented as mean ± standard deviation and compared using Student’s *t*-test. Non-normally distributed variables were presented as median ± interquartile range (IQR) and compared using the Wilcoxon rank-sum test. Categorical variables were presented as percentages and compared using the chi-square test. To assess the significance of differences among groups stratified by quartiles of dietary zinc intake, the Kruskal-Wallis test or one-way analysis of variance was used.

The association between dietary zinc intake and *Helicobacter pylori* (Hp) infection was examined using multivariate logistic regression. Following NHANES analytic guidelines, the sum of dietary zinc intake and zinc supplementation was considered as the total zinc intake. Odds ratios (ORs) and 95% confidence intervals (CIs) were calculated to evaluate the relationship between dietary zinc intake and *Helicobacter pylori* (Hp) infection. Dietary zinc intake was analyzed both as a continuous and categorical variable in the multivariate logistic regression analysis. Three models were used for the logistic regression analysis. Model 1 included no variables, Model 2 included age, gender, marital status, poverty-income ratio, and education, and Model 3 included additional variables such as body mass index, cardiovascular disease, smoking, alcohol consumption, diabetes, and serum indicators (CRP, albumin, total cholesterol, and creatinine). Tests for trend were conducted by including the median value of each quartile as a continuous variable in the models.

Furthermore, in order to address the non-linear association between dietary zinc intake and *Helicobacter pylori* (Hp) infection, we employed a Generalized Additive Model with smooth curve fitting, specifically utilizing the penalized spline method. Additionally, the determination of the inflection point was accomplished through the application of a two-piecewise logistic model and recursive algorithm. A significance level of *p* < 0.05 was considered statistically significant. Subgroup analyses were performed using stratified logistic regression, and the likelihood ratio test was employed to examine interactions between different subgroups. Wald-type intervals were generated on the log-odds scale to evaluate categorical variables. Subgroup analyses were conducted based on age, sex, education, and marital status. Heterogeneity across subgroups was tested using the Wald statistics for cross-product terms of trend variables and subgroup membership.

Finally, we use statistical program, G*Power and R packages(“pwr”) to calculate the sample size requirement and statistical power analysis of this study. After calculation, the power analysis with the population sample size of 2,884 showed a > 99% statistical study power with a *p* value<0.05. The power calculation showed sufficient results for this sample size.

All statistical analyses were carried out using the R statistical software package, version 4.1.1 (R Foundation for Statistical Computing, Vienna, Austria), G*Power and Free Statistics software version 1.7. A significance level of *p* < 0.05 (two-sided) was considered statistically significant. The reporting of this cross-sectional study followed the Strengthening the Reporting of Observational Studies in Epidemiology (STROBE) statement guidelines.

## Results

### Baseline characteristics of selected participants

Table 1 presents the characteristics of the study population. There were 1,281 participants with *Helicobacter pylori* infection, and 47.8% were male. The mean age was 49.5 years old. Compared to the *Helicobacter pylori* antibody negative group, the *Helicobacter pylori* antibody positive group was found to be older, have lower education and income levels, higher BMI, higher prevalence of smoking and diabetes, lower serum albumin level, higher incidence of heart failure, and lower dietary intake of zinc. The proportion of non-drinkers was higher in the *Helicobacter pylori* antibody positive group than in the negative group.

**Table 1:** The baseline characteristics and demographic characteristics of the study population (*N* = 2,884).

Variables	Total (*n* = 2,884)	*Helicobacter pylori* seronegative (*n* = 1,603)	*Helicobacter pylori* seropositivity (*n* = 1,281)	*p* value
Sex, *n* (%)				0.144
Male	1,379 (47.8)	747 (46.6)	632 (49.3)	
Female	1,505 (52.2)	856 (53.4)	649 (50.7)	
Age, Mean ± SD	49.5 ± 18.6	46.9 ± 18.7	52.8 ± 17.9	<0.001
Education, *n* (%)				<0.001
Below high school	1,048 (36.3)	343 (21.4)	705 (55)	
High school	650 (22.5)	403 (25.1)	247 (19.3)	
Above high school	1,186 (41.1)	857 (53.5)	329 (25.7)	
Marital status, *n* (%)				0.158
Living alone	1,063 (36.9)	609 (38)	454 (35.4)	
Married or living with a partner	1821 (63.1)	994 (62)	827 (64.6)	
Poverty-income ratio, *n* (%)				< 0.001
<1.3	865 (30.0)	352 (22)	513 (40)	
≥1.3	2019 (70.0)	1,251 (78)	768 (60)	
Body mass index, *n* (%)				0.006
<25	899 (31.2)	539 (33.6)	360 (28.1)	
25–30	1,033 (35.8)	552 (34.4)	481 (37.5)	
>30	952 (33.0)	512 (31.9)	440 (34.3)	
Smoke status, *n* (%)				0.028
Yes	1,377 (47.7)	736 (45.9)	641 (50)	
No	1,507 (52.3)	867 (54.1)	640 (50)	
Alcohol status, *n* (%)				0.008
Yes	1950 (67.6)	1,117 (69.7)	833 (65)	
No	934 (32.4)	486 (30.3)	448 (35)	
Diabetes, *n* (%)				< 0.001
Yes	262 (9.1)	103 (6.4)	159 (12.4)	
No	2,622 (90.9)	1,500 (93.6)	1,122 (87.6)	
Serum indicators				
Albumin, Mean ± SD	44.2 ± 3.4	44.3 ± 3.6	44.0 ± 3.2	0.007
Total cholesterol, Mean ± SD	198.9 ± 39.7	198.1 ± 39.3	199.9 ± 40.1	0.225
Creatinine, Mean ± SD	0.8 ± 0.6	0.8 ± 0.6	0.8 ± 0.6	0.639
CRP, Mean ± SD	0.5 ± 0.9	0.5 ± 1.1	0.5 ± 0.8	0.373
Cardiovascular disease				
Heartfailure, *n* (%)				0.017
Yes	76 (2.6)	32 (2)	44 (3.4)	
No	2,808 (97.4)	1,571 (98)	1,237 (96.6)	
Coronary disease *n* (%)				0.611
Yes	109 (3.8)	58 (3.6)	51 (4)	
No	2,775 (96.2)	1,545 (96.4)	1,230 (96)	
Angina, *n* (%)				0.059
Yes	111 (3.8)	52 (3.2)	59 (4.6)	
No	2,773 (96.2)	1,551 (96.8)	1,222 (95.4)	
Heart attack, *n* (%)				0.057
Yes	115 (4.0)	54 (3.4)	61 (4.8)	
No	2,769 (96.0)	1,549 (96.6)	1,220 (95.2)	
Dietary intake				
ZINC, Mean ± SD	11.1 ± 7.6	11.6 ± 7.9	10.4 ± 7.3	< 0.001

%, weighted proportion; Hp, *Helicobacter pylori*; CRP, C-reactive protein. Cardiovascular disease (heartfailure, coronary heart disease, angina, heart attack, and stroke). *p* values of multiple comparisons were corrected by the False Discovery Rate method.

### Association between dietary zinc intake and *Helicobacter pylori* seropositivity

Significant differences in factors between zinc intake and *Helicobacter pylori* (Hp) were revealed through univariate analysis, as demonstrated by fold changes ranging from 0.3 to 1.3 (Supplementary Table S1). Age, education, Poverty-Income Ratio (PIR), Body Mass Index (BMI), smoking, alcohol consumption, presence of diabetes, albumin levels, heart failure, and zinc intake all exhibited significant differences between the two groups (Supplementary Table S1).

The association between zinc intake and *Helicobacter pylori* seropositivity was examined using continuous and categorical variables, as shown in Table 2. When zinc intake was analyzed as a continuous variable, each unit increase in zinc intake corresponded to a reduced incidence of *Helicobacter pylori* seropositivity. The odds ratios (OR) for model 1, model 2, and model 3 were 0.98 (95% CI 0.97–0.99), 0.99 (95% CI 0.98–1), and 0.99 (95% CI 0.98–1), respectively.

**Table 2:** Multivariable logistic regression to assess the association of zinc intake with *Helicobacter pylori* seropositivity.

Model	Zinc continuity		Quartile								*p* for trend
			Q1 (<5.97)		Q2 (5.97–8.92)		Q3 (8. 92–13.16)		Q4 (>13.16)		
	OR 95CI	*p* value	OR_95CI	*p* value	OR_95CI	*p* value	OR_95CI	*p* value	OR_95CI	*p* value	*p* value
Model 1	0.98 (0.97 ~ 0.99)	<0.001	1 (Ref)		0.65 (0.53 ~ 0.8)	<0.001	0.68 (0.56 ~ 0.84)	<0.001	0.56 (0.45 ~ 0.69)	<0.001	<0.001
Model 2	0.99 (0.98 ~ 1)	0.084	1 (Ref)		0.71 (0.57 ~ 0.89)	0.003	0.82 (0.65 ~ 1.03)	0.085	0.73 (0.58 ~ 0.93)	0.01	0.039
Model 3	0.99 (0.98 ~ 1)	0.06	1 (Ref)		0.7 (0.56 ~ 0.88)	0.003	0.8 (0.64 ~ 1.01)	0.064	0.72 (0.57 ~ 0.91)	0.007	0.027

Model 1: No adjustment. Model 2: Adjusted for age, gender, marital status, poverty-income ratio, and education. Model 3: Adjusted for age, gender, marital status, poverty-income ratio, education, body mass index, cardiovascular disease (heartfailure, coronary heart disease, and angina, heart attack), smoking, alcohol, diabetes, and serum indicators (CRP, albumin, total cholesterol, and creatinine). Hp, *Helicobacter pylori*; CRP, C-reactive protein; CI, confidence interval; OR, odds ratios; Ref, reference.

Furthermore, when zinc intake was assessed as a categorical variable, individuals in the fourth quartile (Q4) of zinc intake had a significantly lower incidence of *Helicobacter pylori* seropositivity compared to those in the first quartile (Q1). The odds ratio (OR) for this comparison was 0.72 (95% CI 0.57–0.91), after adjusting for various covariates including age, gender, marital status, Poverty-Income Ratio (PIR), education, Body Mass Index (BMI), cardiovascular disease (heart failure, coronary heart disease, angina, heart attack), smoking, alcohol consumption, diabetes, and serum indicators (CRP, albumin, total cholesterol, creatinine). Importantly, the overall trend was found to be statistically significant (*p* < 0.05) using the trend test.

### Dose–response relationships

**Figure 2:**
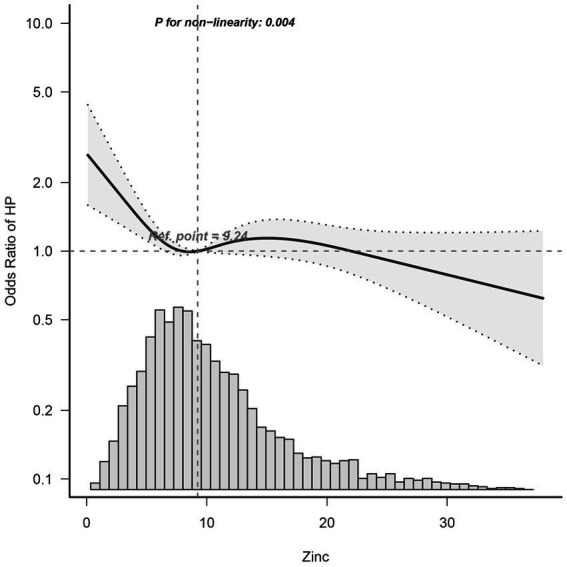
Dose–Response Relationship between dietary zinc intake and *Helicobacter pylori* seropositivity odds ratio. Solid and dashed lines represent the predicted value and 95% confidence intervals. Adjusted for age, gender, marital status, poverty-income ratio, education, body mass index, cardiovascular disease (heartfailure, coronary heart disease, angina, and heart attack), smoking, alcohol, diabetes, and serum indicators (CRP, albumin, total cholesterol, and creatinine). Only 99% of the data is shown. Hp, *Helicobacter pylori*; CI, confidence interval; OR, odds ratios; Ref, reference.

Dietary zinc intake was considered a continuous variable in this study. Utilizing multivariable-adjusted restricted cubic spline analysis, a distinct “L-shaped” association between dietary zinc intake and *Helicobacter pylori* seropositivity was observed (*p* for non-linearity = 0.004) (see Figure 2). By integrating the graphical interpretation with clinical significance, the optimal inflection point for zinc intake was determined to be 24.95 mg/day. In instances where zinc intake was less than 24.925 mg/day, each 1 mg/day increase in zinc intake correlated with a 1.8% reduction in the risk of *Helicobacter pylori* seropositivity (OR, 95% CI: 0.982 [0.965–0.999]; *p* = 0.0346). However, this association was not evident when zinc intake was equal to or exceeded 24.925 mg/day (OR, 95% CI: 1.026 [0.968–1.087]; *p* = 0.3868), as demonstrated in Table 3.

**Table 3:** Threshold effect analysis of relationship of zinc intake and *Helicobacter pylori* seropositivity.

	Zinc	
	Adjusted OR_95CI	*p* value
Two model		
Zinc intake < 24.925	0.982 (0.965 ~ 0.999)	0.0346
Zinc intake ≥ 24.925	1.026 (0.968 ~ 1.087)	0.3868
Non-linear test 1		0.048
Non-linear test 2		0.005

Adjusted for age, gender, marital status, poverty-income ratio, education, body mass index, cardiovascular disease (heartfailure, coronary heart disease, angina, and heart attack), smoking, alcohol, diabetes, and serum indicators (CRP, albumin, total cholesterol, and creatinine). Hp, *Helicobacter pylori*; CRP, C-reactive protein; CI, confidence interval; OR, odds ratios; Ref, reference.

### Subgroup analysis

**Figure 3:**
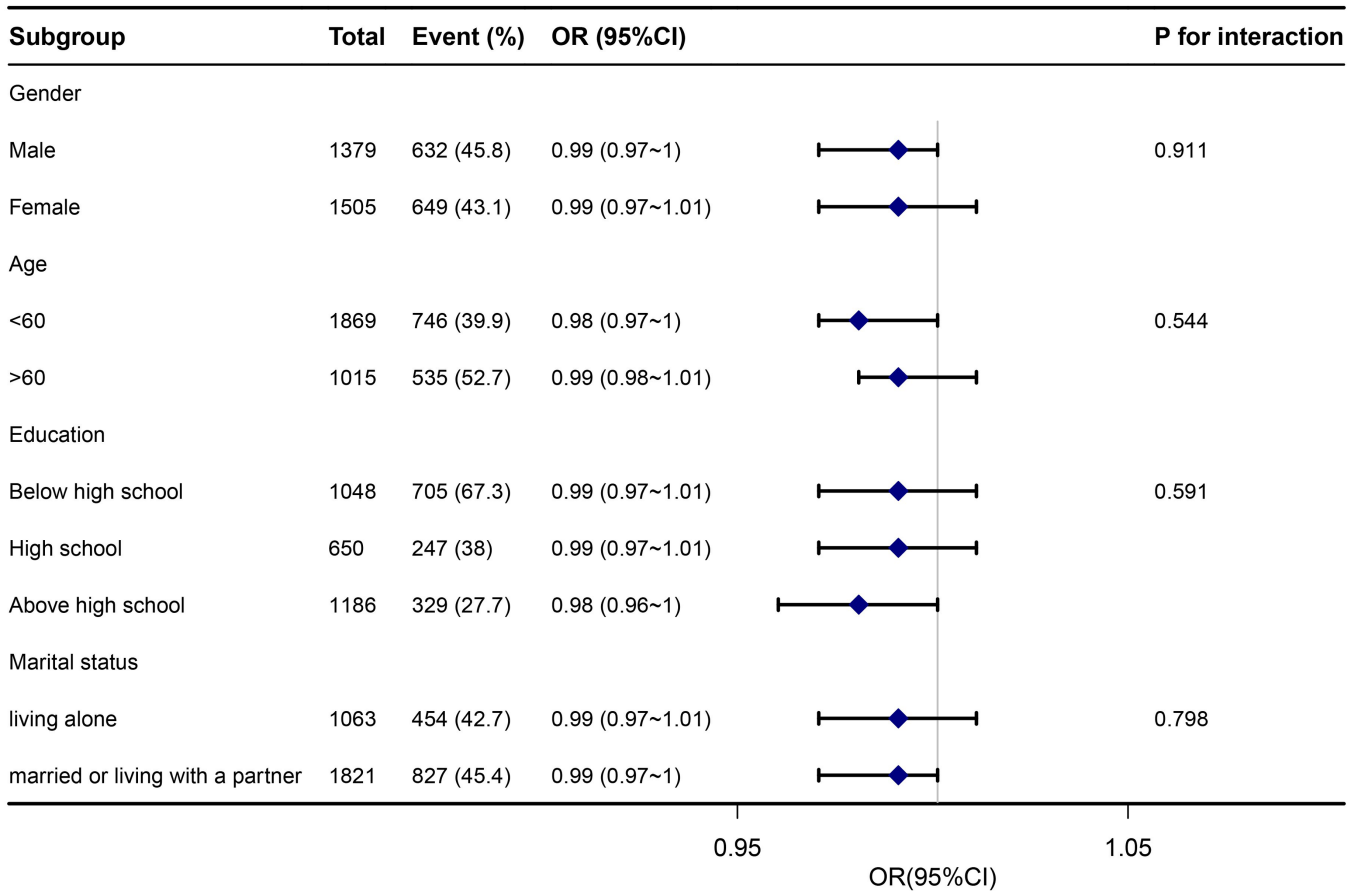
Subgroup analysis association between dietary zinc intake and *Helicobacter pylori* seropositivity. Adjusted for age, gender, marital status, poverty-income ratio, education, body mass index, cardiovascular disease (heartfailure, coronary heart disease, angina, and heart attack), smoking, alcohol, diabetes, and serum indicators (CRP, albumin, total cholesterol, and creatinine). Hp, *Helicobacter pylori*; CI, confidence interval; OR, odds ratios; Ref, reference.

To further investigate the influence of other risk factors on the correlation of zinc intake with *Helicobacter pylori* seropositivity, subgroup analyses were carried on according to the following stratification variables: age, sex, education, and marital status. The results of subgroup analyses and interactions were summarized in Figure 3. Subgroup analysis results were in concordance with those of the multivariable logistic regression analysis. Subgroup analysis results were in concordance with those of the multivariable logistic regression analysis.

## Discussion

This cross-sectional study, which analyzed participants who underwent health examination, elucidated an L-shaped correlation between zinc intake and the presence of *Helicobacter pylori* antibodies. Notably, the inflection point of this relationship was estimated to be around 24.925 mg/day. Moreover, both stratified and sensitivity analyses consistently supported the durability of the association between zinc intake and *Helicobacter pylori* seropositivity.

To the best of our current knowledge, this study represents the initial attempt to examine the correlation between dietary intake of zinc and seropositivity for *Helicobacter pylori* through a cross-sectional approach. Prior investigations pertaining to this subject matter have been rather limited in scope. Nurinnisa’s research indicated that children infected with *H. pylori* exhibit significantly higher serum zinc concentrations compared to their non-infected counterparts, without any discernible variations observed in copper and magnesium levels between the two groups (34). Conversely, Anla’s findings did not reveal any significant disparities in the levels of calcium, magnesium, iron, zinc, selenium, and copper between the infected and non-infected groups (35). A recent study has also identified a noteworthy correlation between seropositivity for *H. pylori* and vitamin D deficiency among children aged 6–36 months. This discovery could potentially contribute to the diagnosis, treatment, and monitoring of both vitamin D deficiency and *H. pylori* infection within this age group (36). Our study aims to address a significant gap in the current understanding of the association between dietary zinc intake and seropositivity for *Helicobacter pylori*. Specifically, our findings shed light on the potential relationship between zinc deficiency and susceptibility to *Helicobacter pylori* seropositivity, an aspect that has not been extensively explored until now.

Although the exact mechanism underlying the inverse relationship between dietary zinc intake and *Helicobacter pylori* seropositivity has yet to be fully elucidated, our findings are supported by available evidence and demonstrate biological plausibility.

One proposed mechanism to explain the association between zinc intake and *Helicobacter pylori* seropositivity is the potential role of zinc in maintaining a healthy gut microbiota, which may contribute to a decreased risk of infection. Research suggests that both the host and the resident gut microbiota require zinc and compete for its availability (37). Additionally, deficiencies in micronutrients, including zinc, can have significant short-and long-term health implications, and reduced dietary intake or impaired absorption can lead to decreased bioavailability (38). Furthermore, alterations in the gut microbiota associated with *H. pylori* infection have been implicated in the disruption of the intestinal mucosal barrier and the development of early-stage colorectal carcinoma (39, 40). Characterizing the differences in bacterial and fungal composition could provide valuable insights for the development of novel biomarkers and management strategies.

Another potential mechanism that may influence the association between zinc and *Helicobacter pylori* seropositivity involves Toll-like receptors. Recent studies have revealed a significant correlation between zinc status and Toll-like receptors in the body, which could impact infection and immune function in children (41). Moreover, Toll-like receptors play a crucial role in *Helicobacter pylori* infection (42). As part of the innate immune system, Toll-like receptors act as the first line of defense against bacterial invasion. They belong to the family of pattern recognition receptors (PRRs), and their activation leads to the production of inflammatory cytokines, chemokines, antigen-presenting molecules, and costimulatory molecules. Numerous investigations have explored the relationship between Toll-like receptors and *Helicobacter pylori*-associated diseases (43).

Another notable contribution of this paper is the identification of a relationship between zinc intake and *Helicobacter pylori* seropositivity, characterized by a L-shaped curve, indicating a non-linear association and a potential threshold effect. Additionally, subgroup and sensitivity analyses have demonstrated the overall stability of the results. Furthermore, it is concerning to observe that a considerable proportion, up to 17.3%, of the global population is susceptible to zinc deficiency, with over 25% of countries exhibiting diets deficient in zinc, thereby posing a high risk of this deficiency (44). Among various dietary supplements, organic zinc, specifically zinc lactate, has exhibited the most effective protective effect (45). Consequently, our study holds significant implications for clinical practice, research endeavors, and health policy. These findings emphasize the necessity for heightened attention to zinc intake in future studies.

The present study utilizes a methodology that offers a number of noteworthy advantages. Firstly, it represents the initial investigation to explore the correlation between Zinc Intake and *Helicobacter pylori* seropositivity among individuals residing in the United States. Secondly, a smoothing function analysis was employed to address potential contingencies in the data analysis, thereby providing a clearer understanding of the relationship between Zinc Intake and *Helicobacter pylori* seropositivity. Additionally, this study evaluated dietary zinc intake as categorical variables, reducing contingencies and bolstering the robustness of the results. Nevertheless, certain limitations should be acknowledged. Firstly, the cross-sectional design of the study precludes the establishment of a temporal relationship between dietary Zinc Intake and *Helicobacter pylori* seropositivity. Consequently, future prospective cohort studies are necessary to validate our findings. Moreover, the reliance on self-reported data from interviews and 24-h dietary assessments in this study introduces the potential for recall and reporting bias, despite the validation of these assessment tools in other research. Furthermore, despite its prior utilization in other studies, the application of the IgG-based enzyme-linked immunosorbent assay (ELISA) in our investigation is constrained by the limited availability of relevant tests in the NHANES database. This assay lacks the capability to distinguish recent infections from past ones and may potentially overlook acute cases of *H. pylori* infection. Moving forward, we intend to incorporate additional indicators to enhance the measurement of *Helicobacter pylori* infection in our future study (46–48). Lastly, the study participants were exclusively derived from the American population, thereby restricting the generalizability of the findings beyond this demographic. It is crucial to consider this aspect when extrapolating the results to other populations. Given these limitations, well-designed multicenter controlled trials are indispensable to substantiate our findings.

## Conclusion

In summary, the findings of this study suggest a potential inverse relationship between dietary zinc intake and the prevalence of *Helicobacter pylori* seropositivity. Among adults in the NHANES dataset, a non-linear association resembling an L-shape was observed between dietary zinc intake and *Helicobacter pylori* seropositivity, with an inflection point estimated at approximately 24.925 mg/day. Nevertheless, it is important to note that further substantiation of these results necessitates the inclusion of extensive prospective research studies with prolonged follow-up periods.

## Data availability statement

The datasets presented in this study can be found in online repositories. The names of the repository/repositories and accession number(s) can be found at: https://www.cdc.gov/nchs/nhanes/index.htm.

## Ethics statement

The NCHS Research Ethics Review Committee reviewed and approved the NHANES study protocol. The studies were conducted in accordance with the local legislation and institutional requirements. The participants provided their written informed consent to participate in this study. Written informed consent was obtained from the individual(s) for the publication of any potentially identifiable images or data included in this article.

## Author contributions

KZ is the first author of article. YH, FMG, and ZXG are responsible for the concept and design of the study. JYZ, JGC, BWC, and MG explain the analysis. ZYH, XQY, TYC, YFG, JYX, RH, and TZL are responsible for data recovery. KZ and TZL are the article and corresponding authors. All authors contributed to the article and approved the submitted version.

## Conflict of interest

The authors declare that the research was conducted in the absence of any commercial or financial relationships that could be construed as a potential conflict of interest.

## Publisher’s note

All claims expressed in this article are solely those of the authors and do not necessarily represent those of their affiliated organizations, or those of the publisher, the editors and the reviewers. Any product that may be evaluated in this article, or claim that may be made by its manufacturer, is not guaranteed or endorsed by the publisher.

## Supplementary material

The Supplementary material for this article can be found online at: https://www.frontiersin.org/articles/10.3389/fnut.2023.1243908/full#supplementary-material

## Footnotes

^1^ https://www.cdc.gov/nchs/nhanes/index.htm
