# Supplementary material for: Association between dietary zinc intake and Helicobacter pylori seropositivity in US adults: National Health and Nutrition Examination Survey
Source: Front Nutr. 2023 Sep 21;10:1243908. doi: 10.3389/fnut.2023.1243908 (PMC10551451; doi:10.3389/fnut.2023.1243908)
Supplement: Supplementary file 1 [file Table_1.docx]

| supplement Table Univariate logistic regression to assess the association of zinc intake with Helicobacter pylori seropositivity | | |
| --- | --- | --- |
| Variable | OR_95CI | P value |
| Male | 1(Ref) | |
| Female | 0.9 (0.77~1.04) | 0.144 |
| Age | 1.02 (1.01~1.02) | <0.001 |
| Below high school | 1(Ref) | |
| High school | 0.3 (0.24~0.37) | <0.001 |
| Above high school | 0.19 (0.16~0.22) | <0.001 |
| Marital status | 1.12 (0.96~1.3) | 0.158 |
| PIR＜1.3 | 1(Ref) | |
| PIR≥1.3 | 0.42 (0.36~0.5) | <0.001 |
| BMI＜25 | 1(Ref) | |
| BMI(25-30) | 1.3 (1.09~1.56) | 0.004 |
| BMI＞30 | 1.29 (1.07~1.55) | 0.007 |
| Smoke status | 1(Ref) | |
| No Smoke status | 0.85 (0.73~0.98) | 0.028 |
| Alcohol status | 1(Ref) | |
| No Alcohol status | 1.24 (1.06~1.45) | 0.008 |
| Diabetes | 1(Ref) | |
| No Diabetes | 0.48 (0.37~0.63) | <0.001 |
| Albumin | 0.97 (0.95~0.99) | 0.007 |
| Total cholesterol | 1 (1~1) | 0.225 |
| Creatinine | 0.97 (0.86~1.1) | 0.64 |
| CRP | 1.04 (0.96~1.12) | 0.38 |
| Heart failure | 1(Ref) | |
| No Heart failure | 0.57 (0.36~0.91) | 0.018 |
| Coronary disease | 1(Ref) | |
| No Coronary disease | 0.91 (0.62~1.33) | 0.612 |
| Angina | 1(Ref) | |
| No Angina | 0.69 (0.47~1.02) | 0.06 |
| Heart attack | 1(Ref) | |
| No heart attack | 0.7 (0.48~1.01) | 0.059 |
| ZINC | 0.98 (0.97~0.99) | <0.001 |
| Abbreviations: %, weighted proportion.; | | |
| Hp：Helicobacter pylori，CRP：C-reactive protein | | |
| Cardiovascular disease(heartfailure, coronary heart disease, angina, heart attack, stroke) | | |
| CI:confidence interval；OR：odds ratios，Ref:reference | | |
